# Genome-Scale Transcriptome Analysis of the Desert Shrub *Artemisia sphaerocephala*

**DOI:** 10.1371/journal.pone.0154300

**Published:** 2016-04-26

**Authors:** Lijing Zhang, Xiaowei Hu, Xiumei Miao, Xiaolong Chen, Shuzhen Nan, Hua Fu

**Affiliations:** State Key Laboratory of Grassland Agro-ecosystems, College of Pastoral Agriculture Science and Technology, Lanzhou University, Lanzhou, China; CSIR-National Botanical Research Institute, INDIA

## Abstract

**Background:**

*Artemisia sphaerocephala*, a semi-shrub belonging to the *Artemisia* genus of the *Compositae* family, is an important pioneer plant that inhabits moving and semi-stable sand dunes in the deserts and steppes of northwest and north-central China. It is very resilient in extreme environments. Additionally, its seeds have excellent nutritional value, and the abundant lipids and polysaccharides in the seeds make this plant a potential valuable source of bio-energy. However, partly due to the scarcity of genetic information, the genetic mechanisms controlling the traits and environmental adaptation capacity of *A*. *sphaerocephala* are unknown.

**Results:**

Here, we present the first in-depth transcriptomic analysis of *A*. *sphaerocephala*. To maximize the representation of conditional transcripts, mRNA was obtained from 17 samples, including living tissues of desert-growing *A*. *sphaerocephala*, seeds germinated in the laboratory, and calli subjected to no stress (control) and high and low temperature, high and low osmotic, and salt stresses. *De novo* transcriptome assembly performed using an Illumina HiSeq 2500 platform resulted in the generation of 68,373 unigenes. We analyzed the key genes involved in the unsaturated fatty acid synthesis pathway and identified 26 *A*. *sphaerocephala fad2* genes, which is the largest *fad2* gene family reported to date. Furthermore, a set of genes responsible for resistance to extreme temperatures, salt, drought and a combination of stresses was identified.

**Conclusion:**

The present work provides abundant genomic information for functional dissection of the important traits of *A*. *sphaerocephala* and contributes to the current understanding of molecular adaptive mechanisms of *A*. *sphaerocephala* in the desert environment. Identification of the key genes in the unsaturated fatty acid synthesis pathway could increase understanding of the biological regulatory mechanisms of fatty acid composition traits in plants and facilitate genetic manipulation of the fatty acid composition of oil crops.

## Introduction

China has the largest desertification area in the world, with a total desertified land area of 2,623,700 km^2^ comprising 27.33% of the national territory by the end of 2009 [1]. With great efforts in reforestation and revegetation, desertification in China has been reversed, as shown by the annual decrease in the desertified land area of 1,375 km^2^ over the last 10 years [2]. Desert plants are highly adaptable to adverse environmental conditions [3,4] and are attracting increasing research interest regarding the genetic basis of their unique adaptation and survival abilities [5–9]. These plants are also uniquely economically valuable [10].

*Artemisia sphaerocephala*, a dicotyledonous perennial semi-shrub belonging to the *Artemisia* genus of the *Compositae* family, is distributed widely in the provinces of Gansu, Ningxia, Shanxi, and Xinjiang, as well as in Inner Mongolia, northwestern China [11,12]. This shrub is one of the most important pioneer plants in moving and semi-stable sand dunes in the deserts and steppes, protecting rangelands from wind erosion and playing a vital role in maintaining desert ecosystem stability [13]. *Artemisia sphaerocephala* can be consumed as food by humans; early herders consumed the seeds during ancient times. In addition, it is rich in linoleic acid, and the oil content of its seeds is 21.5% [14], with unsaturated fatty acids accounting for 91% of the total fatty acids and linoleic acid accounting for 81% of the total unsaturated fatty acids [15]. Further, the total fatty acid concentration in *A*. *sphaerocephala* leaves is 11.42 mg/g, and linoleic acid accounts for 22.31% of the total fatty acids [16]. Linoleic acid is one of the main polyunsaturated fatty acids in cell membranes, and an increase in its accumulation is thought to aid in maintenance of membrane fluidity and cellular integrity during stress [17–19]. In addition, linoleic acid is an essential fatty acid for mammals [20], and it is the precursor of conjugated linoleic acid (CLA), which is generated in the stomachs of ruminant animals as well as those of several non-ruminant animals [21]. CLA has significant protective functions against obesity [22,23], cancer [24], inflammation [25], and diabetes [26], and it is beneficial for energy metabolism [23]. Thus, there is great interest in increasing the amount of CLA in the human food supply because of its potential benefits to human health [27]. *Artemisia sphaerocephala* seeds are also rich in polysaccharides (up to 35% of the seed dry mass [12]), which are a type of raw material used to produce ethanol [28]. Due to the large quantities of polysaccharides and lipids in *A*. *sphaerocephala* seeds, this desert shrub has the potential for use as a bio-fuel for diesel engines.

Transcriptome analysis aims to capture an unbiased view of the complete RNA transcript profile of a species, allowing for monitoring of the transcript level of each gene in a given tissue at a given point in the organisms’ life cycle [29]. During recent years, next-generation sequencing technology has been applied in studies of desert plants, such as *Populus euphratica* [6], *Rhazya stricta* [30], and *Ammopiptanthus mongolicus* [31]. Despite its important ecological and economic value, no transcriptome or genomic sequences are currently available for *A*. *sphaerocephala* in the GenBank database. In this paper, we present a *de novo* assembly of the *A*. *sphaerocephala* transcriptome performed using an Illumina HiSeq 2500 platform. Data were collected via sequencing of cDNA libraries of living tissues obtained from shrubs growing in the Alxa Desert. We further analyzed genes related to the unsaturated fatty acid synthesis pathway in this species and constructed a phylogenetic tree of *fad2* genes. This information could improve our understanding of unsaturated fatty acid metabolic pathways in *A*. *sphaerocephala*. We specifically examined the gene expression dynamics of this plant in response to stresses and identified a set of stress-related transcripts. Our findings could shed light on the possible adaptive mechanisms of *A*. *sphaerocephala* in the desert.

## Materials and Methods

### Plant materials

Mature leaves, stems, roots, flowers, flower buds, early developing seeds, mid-developing seeds and mature seeds were collected from *A*. *sphaerocephala* plants growing in the Alxa Desert of Inner Mongolia, northwest China (N: 38°68’, E: 105°61’). The sampling ground is in an uninhabited desert; no specific permission was required and no endangered or protected species were involved in this study. In addition, we collected plant materials in the laboratory, including germinated seeds after 3 days, germinated seeds after 7 days, seedlings and 6 different calli. The calli were cultivated using the method described by Xu and Jia [32]. One month later, the calli were assigned to 5 treatment groups and 1 control group and exposed to the following conditions: a) addition of 100 mM NaCl to the medium to induce salt stress; b) addition of PEG-8000 to the medium to induce osmotic stress of -0.7 MPa and -1.7 MPa [33]; (c) incubation at 40°C and 4°C to induce high- and low-temperature stresses, respectively; and d) normal conditions (untreated control group). After 24 h, all samples were rapidly frozen in liquid nitrogen and stored at -80°C for later RNA extraction.

### RNA extraction

Total RNA was extracted from each tissue using an RNAprep Pure Plant Kit (Tiangen, China). The quantity and quality of total RNA were determined using a NanoDrop ND1000 (Thermo Science, USA), Agilent 2100 Bioanalyzer (Agilent, USA), Qubit 2.0 and gel electrophoresis. At least 20 μg of total RNA was used for cDNA library preparation.

### Library preparation and RNA sequencing

Illumina sequencing was performed following the manufacturer’s instructions. First, oligo(dT) beads were used for enrichment of eukaryotic mRNA. Subsequently, poly(A)^+^ RNA was purified and fragmented into smaller pieces. First-strand cDNA was synthesized with random hexanucleotide primers (random hexamers) using small RNA fragments as templates. Second-strand cDNA was then synthesized in a reaction mixture containing buffer, dNTPs, RNase H and DNA polymerase I. Then, AMPure XP beads were used to purify the cDNA. The purified double-stranded cDNA was end-repaired, and poly(A) tails were added; then, AMPure XP beads were used to select fragments of a particular size. Finally, a sequencing library was constructed by PCR enrichment. After construction of the library, Qubit 2.0 and an Agilent 2100 Bioanalyzer were used to determine the the concentrations and sizes of the inserts. To ensure the high quality of the library, qRT-PCR was performed to measure the effective concentrations of the library reads. Then, high-throughput sequencing was conducted using an Illumina HiSeq 2500 platform. In total, 46.83 million reads were generated, 86.15% of which had a quality score above Q30 (Table 1).

**Table 1:** Summary of transcriptome sequencing results.

Sample	Read Number	Base Number	GC Content	%≥Q30
***Artemisia sphaerocephala***	46,831,604	9,458,445,195	44.00%	86.15%

### *De novo* assembly and assessment

Clean reads were obtained after filtering adaptor sequences and reads with ambiguous “N” bases and those with a base quality score of less than Q30. Further, all sequences smaller than 60 bases were eliminated because such small reads might be sequencing products [34]. The high-quality reads were then assembled into unigenes using Trinity software with a sensitivity similar to methods that rely on genome alignments [35].

### qRT-PCR analysis

Total RNA was extracted from 6 calli exposed to different conditions using RNAprep Pure Plant Kit (Tiangen, China). Then, the RNA was reverse transcribed (Prime-Script RT-PCR Kit, Takara, China) into cDNA to measure the expression of 5 genes that are commonly involved in the responses to PEG-induced drought stress, extreme temperatures and high salinity (the sequences of these 5 genes are provided in S1 Table). qRT-PCR was performed using an MyiQ Single-Color Real-Time PCR Detection System (Bio-Rad, USA) and a SYBR Premix Ex Taq Kit (Takara, China), which is a real-time PCR kit. PCR was performed under the following conditions: 95°C for 30 s, followed by 40 cycles at 95°C for 5 s for denaturation and 60°C for 30 s for annealing and extension. The experiments were repeated three times using independent RNA samples, and actin was used as an internal control. Relative gene expression was calculated with the 2^-ΔΔCt^ method [36]. Synthesis of qRT-PCR primers for the 5 genes were completed by Sangon Biotech Company (Sangon, China), and SPSS software was used for data analysis (P<0.05). Primers information is presented in S2 Table.

## Results and Discussion

### Sequence analysis and assembly

**Fig 1:**
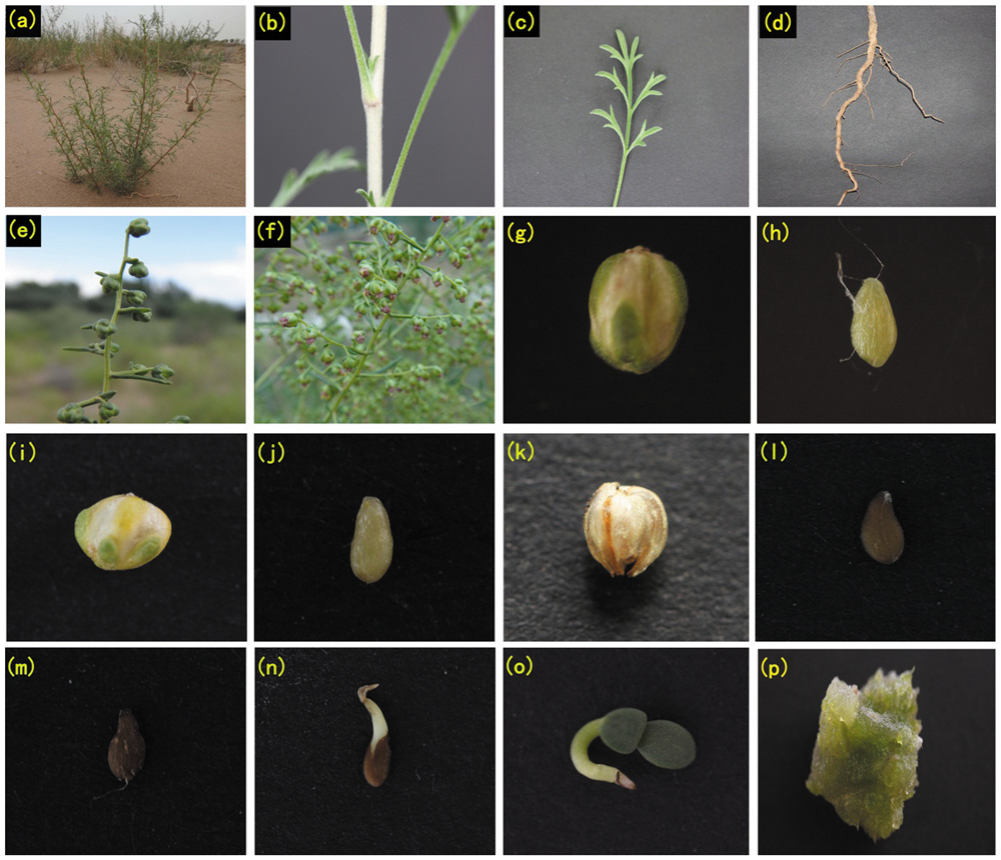
The morphologies of various *Artemisia sphaerocephala* tissues. Note: (a) Whole plant, (b) stem, (c) leaf, (d) root, (e) flower bud, (f) flower, (g) early developing seed husk, (h) early developing seed, (i) mid-developing seed husk, (j) mid-developing seed, (k) mature seed husk, (l) mature seed, (m) germinating seed at 3 days, (n) germinating seed at 7 days, (o) seedling, and (p) callus cell.

To obtain a global and comprehensive overview of the *A*. *sphaerocephala* transcriptome, total RNA was obtained from 17 different plant tissues (the morphologies of the various tissues are shown in Fig 1). Comparable amounts of total RNA from each tissue were mixed. Then, each specimen was sequenced using an Illumina HiSeq 2500 platform. After the reads were subjected to stringent quality assessment and data filtering, 46,831,604 reads (9.46 Gb) with 86.15% Q30 bases remained. A summary of the transcriptome sequencing results is presented in Table 1. The raw high-quality sequence datasets were archived in the National Center for Biotechnology Information (NCBI) Short Read Archive database (SAMN03951130).

**Fig 2:**
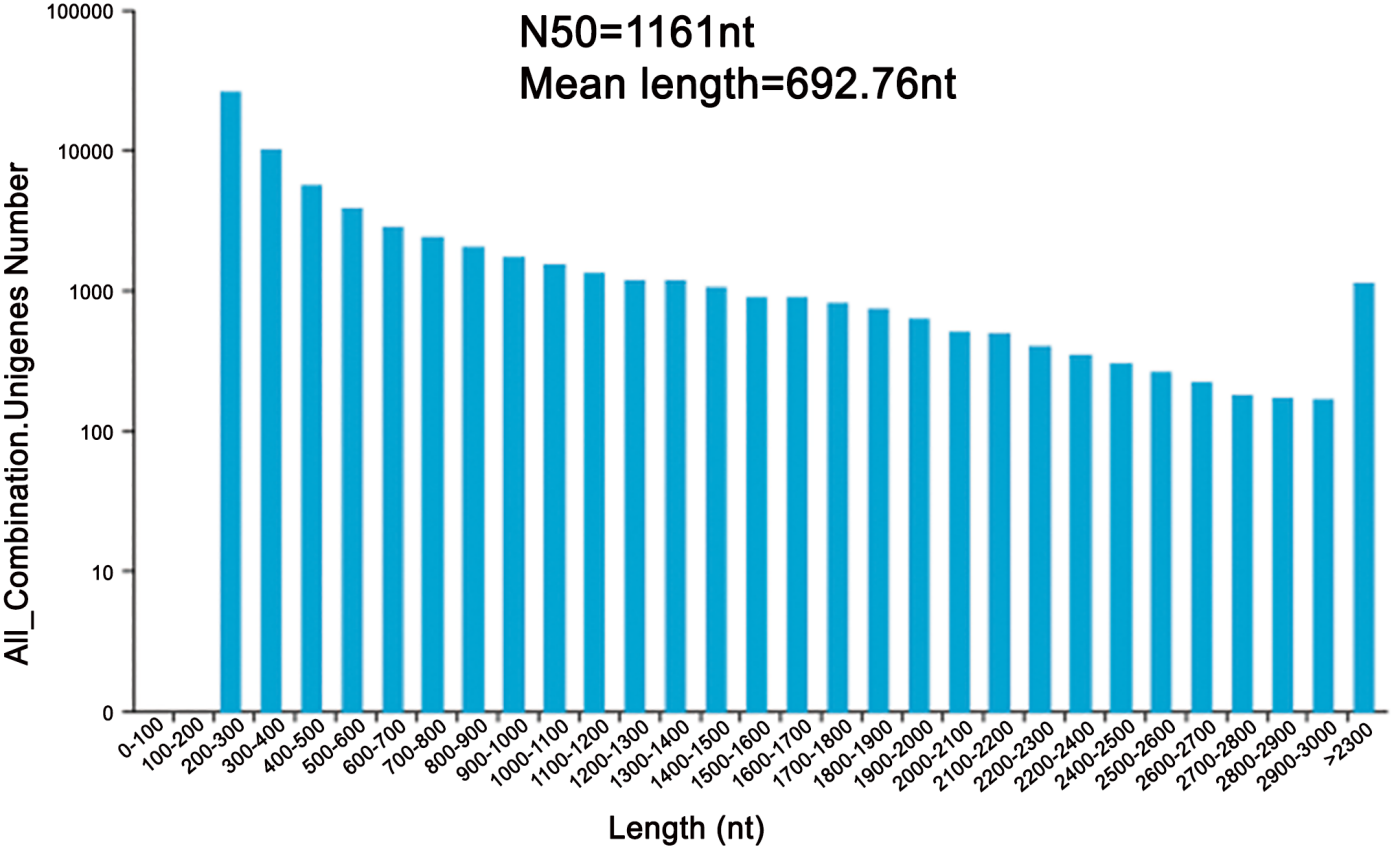
Histogram of length distribution of unigenes.

**Fig 3:**
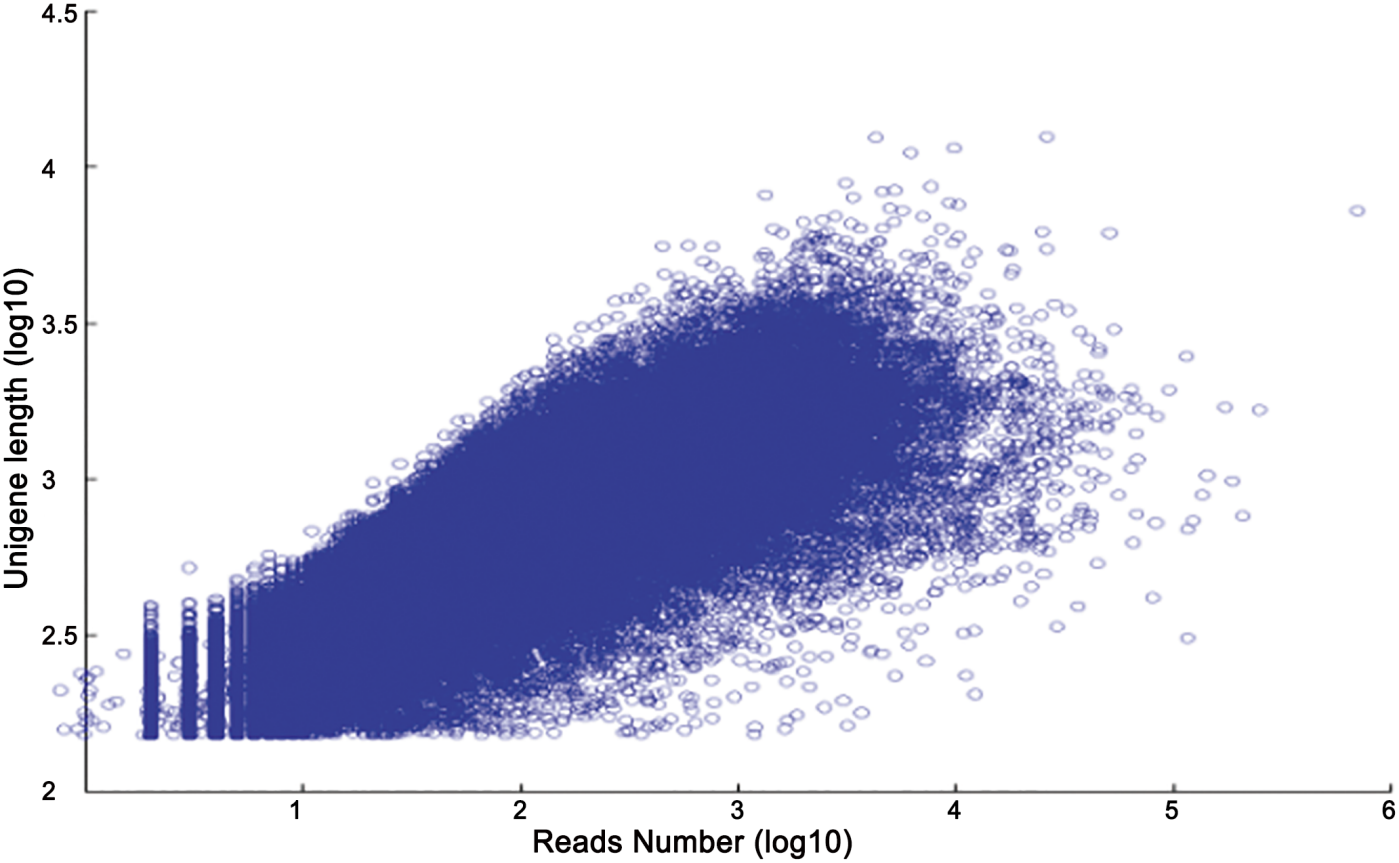
Plot showing the dependence of unigene length on the number of reads assembled into the corresponding unigenes.

All high-quality clean reads were assembled *de novo* with Trinity software [35], as shown in Table 2, which generated 137,060 transcripts with an average length of 884.39 nt and an N50 of 1,403 nt (S1 Fig). After further analyses were performed, 68,373 unigenes with a mean length of 692.76 nt and an N50 of 1,161 nt were obtained (Fig 2). A positive relationship was found between unigene length and the number of reads assembled into the corresponding unigenes, as expected for a randomly fragmented transcriptome (Fig 3). Moreover, open reading frames (ORFs) were predicted using getORF software (http://emboss.sourceforge.net/apps/cvs/emboss/apps/getorf.html), which revealed that the ORFs of 67,917 unigenes (99.33%) had an average length of 444.59 nt and an N50 of 963 nt (S3 Table and S2 Fig). These results indicated that high transcriptomic coverage was achieved for this species.

**Table 2:** Overview of *de novo* sequencing and assembly.

Length Range	Contig	Transcript	Unigene
**200–300**	6,446,268 (99.23%)	34,417 (25.11%)	25,657 (37.53%)
**300–500**	19,666 (0.30%)	27,213 (19.85%)	15,593 (22.81%)
**500–1000**	15,158 (0.23%)	31,877 (23.26%)	12,764 (18.67%)
**1000–2000**	10,786 (0.17%)	31,160 (22.73%)	10,188 (14.90%)
**2000+**	4,323 (0.07%)	12,393 (9.04%)	4,171 (6.10%)
**Total Number**	6,496,201	137,060	68,373
**Total Length**	321,712,475	121,214,445	47,366,348
**N50 Length**	48	1,403	1,161
**Mean Length**	48.14	884.39	692.76

### Functional annotation

All unigenes were aligned to the NCBI non-redundant (Nr) protein database and annotated using Clusters of Orthologous Groups (COG) of protein, gene ontology (GO) terms, the Kyoto Encyclopedia of Genes and Genomes (KEGG) pathway database and the Swiss-Prot protein database, with a BLAST threshold of less than 1E-5 [37]. Out of the 68,373 unigenes, 40,153 (58.70%) significantly matched sequences deposited in the public protein databases (Table 3). Approximately 59.4% of the unigenes had best hits to known plant genes (E-value<1E-50, S3 Fig). Technical limitations, such as sequencing depth and read length, influenced the rate of transcriptomic annotation to some extent [30, 38, 39]. The mean length of the annotated unigenes was longer than that of the un-annotated unigenes (S4 Fig, 868.1683 nt vs. 335.1881 nt), and the expression levels of the annotated unigenes inferred from the fragments per kilobase of transcript per million mapped reads (FPKM) were much higher than those of the un-annotated unigenes (S5 Fig). A possible explanation for the low percentage of annotated unigenes (58.7%) is that the unigenes might be species-specific, which would prevent their annotation.

**Table 3:** Summary of sequence annotation.

Annotated_Databases	Number	300≤length<1,000	length≥1,000	Percentage (%)
**COG**	13,041	4,634	6,008	19.10
**GO**	28,997	11,721	11,042	42.40
**KEGG**	9,148	3,772	3,440	13.40
**Swiss-Prot**	26,508	11,087	10,358	38.80
**Nr**	39,923	16,954	13,760	58.40
**All**	40,153	17,064	13,778	**58.70**

**Fig 4:**
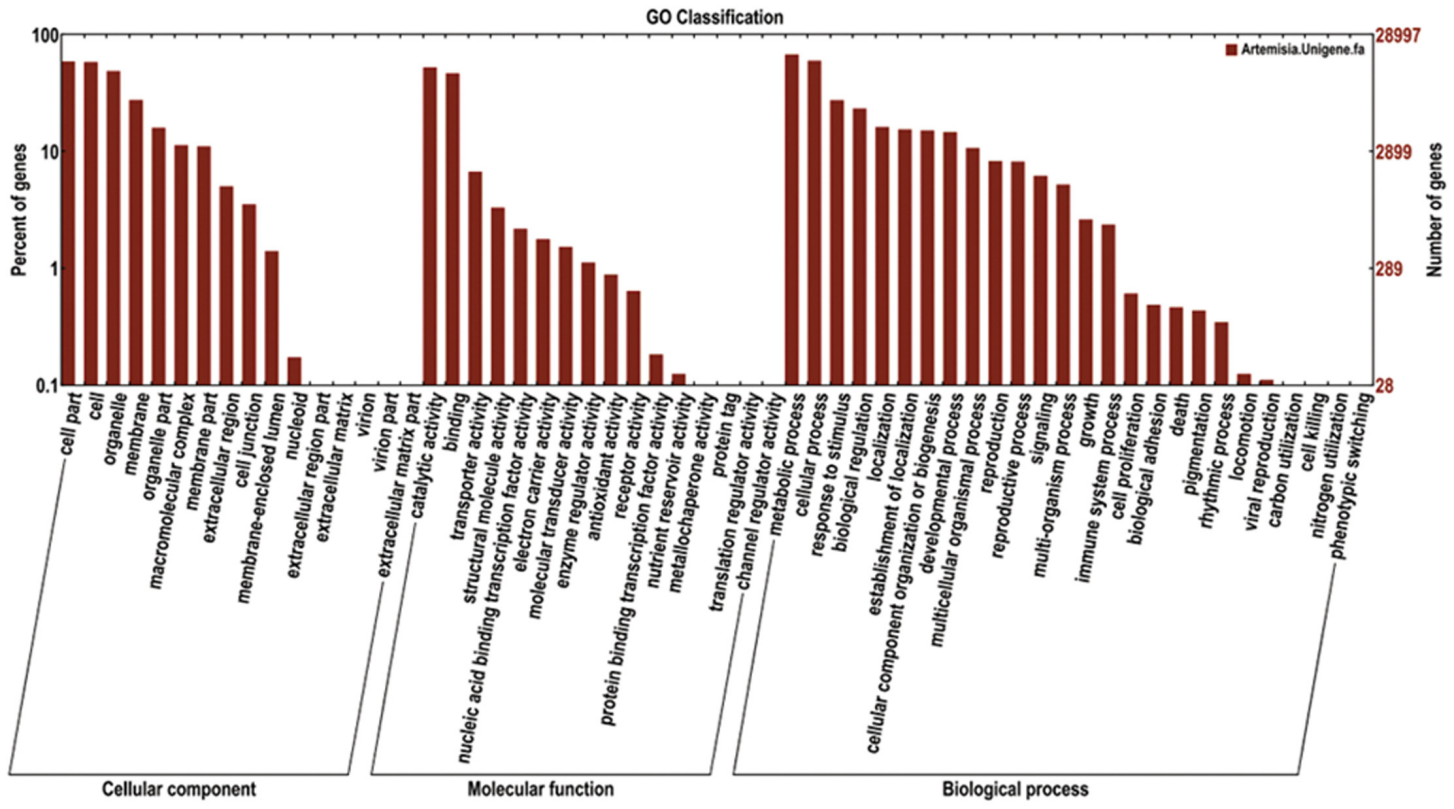
Clusters of Orthologous Group classifications.

**Fig 5:**
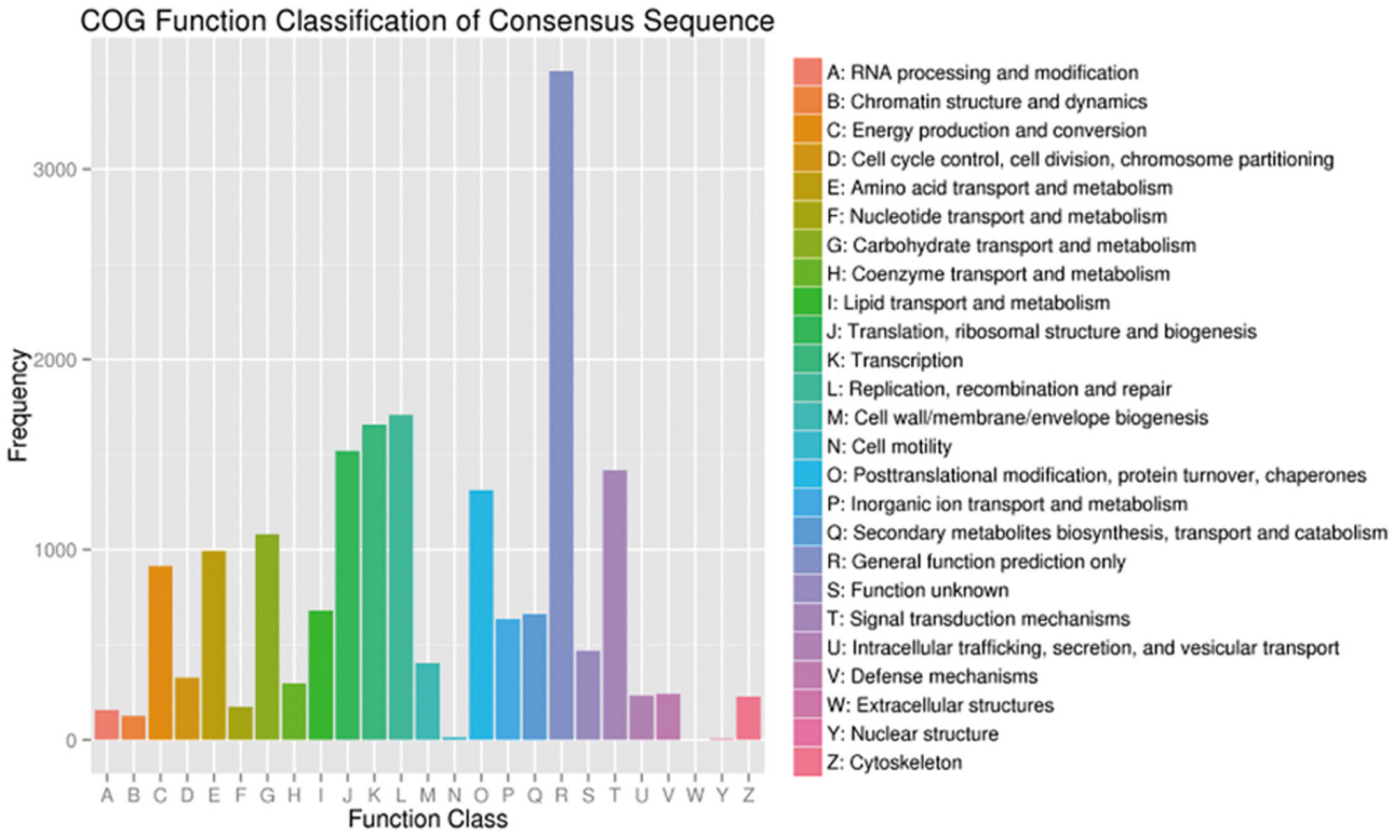
GO categories of the *Artemisia sphaerocephala* unigenes.

A summary of the final *A*. *sphaerocephala* transcriptome annotation results is shown in S4 Table. Annotation of all 68,373 unigenes was performed using the COG database, resulting in classification of 13,041 (19.10%) unigenes into COG categories (Fig 4). Among the 25 COG classifications, the cluster for general function prediction was predominant, followed by replication, recombination and repair. Further, GO annotation, with assignment of the *A*. *sphaerocephala* unigenes to molecular function, biological process and cellular component GO terms, was performed (E-value<1E-5, Fig 5). The top 50 represented GO terms are shown in S6 Fig. A total of 42.4% of the unigenes (28,997) had a significant hit in GO public databases, and 28,997 unigenes were assigned to at least one GO term. The most abundant biological process GO terms were oxidation-reduction process (2,324 unigenes) and protein phosphorylation (935 unigenes). GO terms associated with responses to other environmental factors, such as cadmium ion (717 unigenes), salt (671 unigenes), cold (447 unigenes), abscisic acid (397 unigenes), bacteria (380 unigenes), wounding (332 unigenes) and water deprivation (323 unigenes), were also enriched. The highly enriched biological process GO terms indicated that most of the annotated unigenes were associated with elementary responses to environmental factors. In addition, 22.04% of the unigenes shared over 80% similarity with sequences deposited in the Nr database (S7 Fig).

To identify and screen for the prominent metabolic pathways in *A*. *sphaerocephala*, the acquired unigenes were mapped to metabolic pathways in the KEGG database. In total, 9,148 unigenes were mapped to 117 metabolic pathways. Most unigenes were assigned to 3 pathways, namely Ribosome, Protein processing in endoplasmic reticulum and Oxidative phosphorylation (717, 359 and 338, respectively) (Table 4).

**Table 4:** The top 15 pathways in *Artemisia sphaerocephala*.

	Pathway	Unigene number
**1**	Ribosome	717
**2**	Protein processing in endoplasmic reticulum	359
**3**	Oxidative phosphorylation	338
**4**	Spliceosome	307
**5**	RNA transport	305
**6**	Plant hormone signal transduction	288
**7**	Glycolysis / Gluconeogenesis	284
**8**	Plant-pathogen interaction	236
**9**	Purine metabolism	231
**10**	Starch and sucrose metabolism	189
**11**	Pyruvate metabolism	187
**12**	Ubiquitin-mediated proteolysis	179
**13**	Endocytosis	177
**14**	Ribosome biogenesis in eukaryotes	174
**15**	Amino sugar and nucleotide sugar metabolism	166

### Fatty acid synthesis-related genes in *Artemisia sphaerocephala*

**Fig 6:**
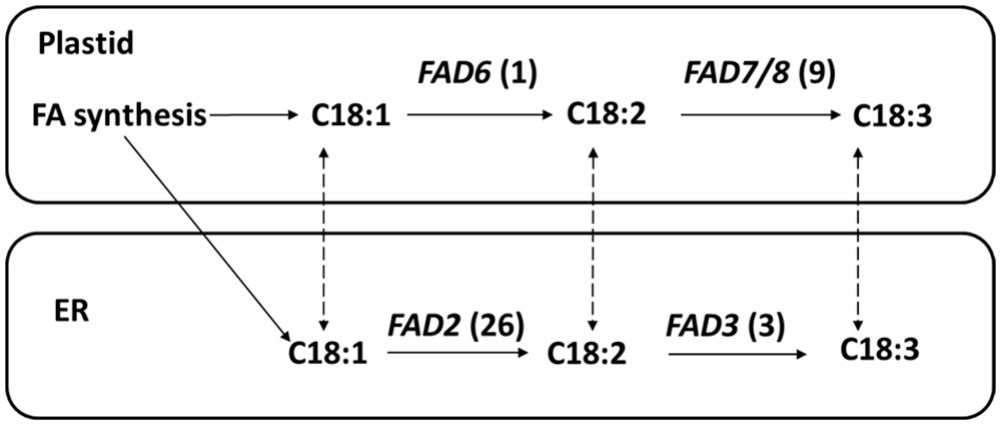
*Artemisia sphaerocephala fad* genes involved in the unsaturated fatty acid synthesis pathway. Note: The numbers in the brackets indicate gene numbers.

Fatty acid compositions vary among different species (Table 5). Linoleic acid (18:2) is the most abundant (80%) fatty acid in *A*. *sphaerocephala* seed oil, and linoleic and linolenic acids in the leaf comprise 22.31% and 46.33% of the total fatty acids, respectively [16]. The pathways for polyunsaturated fatty acid production in plants are generally well understood and have been largely elucidated [40]. C18:1 may be converted to C18:2 in plastids by a membrane-bound fatty acid desaturase called FAD6, or C18:1 may be exported from the plastids to the ER for conversion to C18:2 by a structurally related enzyme called FAD2. In a similar manner, C18:2 may be converted to C18:3 in plastids by the FAD7 or FAD8 enzyme, or it can be exported to the ER for conversion to C18:3 by the FAD3 enzyme [19]. Different species have different fatty acid desaturases (Table 6). In the *A*. *sphaerocephala* transcriptome, 26 putative *fad2s*, 3 putative *fad3s*, 1 putative *fad6* and 9 putative *fad7/fad8s* were identified (Fig 6). With regard to *fad2*, safflower has 11 *fad2s* [41], soybean has 5 [42, 43], cotton [44–47] and rapeseed [48] each have 4, sunflower [49] and peanut [50, 51] each have 3, and *A*. *thaliana* has 1 [52]. Although previous studies have shown that flax has 15 *fad2s* [53–55], only one protein sequence was found in the NCBI database because the base substitutions of 15 *fad2* genes in flax do not result in amino acid changes. We identified 20 *fad2* genes from the *Artemisia annua* transcriptomic data [56]. In this study, we found the largest *fad*2 gene family to date. As shown in Table 5, the linoleic acid concentrations in the leaves and seeds of *A*. *sphaerocephala* are higher than those in other plants, and the large *fad2* gene family may play a prominent role in fatty acid synthesis.

**Table 5:** Fatty acid compositions of different species.

Species	Organ	C18:1	C18:2	C18:3	Reference
***Artemisia sphaerocephala***	Seeds	9.6%	80.0%	0.2%	[15]
	Leaves	5.2%	22.3%	46.3%	[16]
***Carthamus tinctorius***	Seeds	17.0%	70.0%	0.2%	[57]
***Helianthus annuus***	Seeds	24.1%	64.9%	0.2%	[58]
	Leaves	N/A	11.6%	0.7%	[59]
***Glycine max***	Seeds	23.0%	54.0%	8.0%	[60]
	Leaves	1.4%	10.6%	71.3%	[61]
***Gossypium hirsutum***	Seeds	16.0%	53.0%	0.2%	[62]
***Arabidopsis thaliana***	Seeds	13.2%	27.5%	19.2%	[63]
	Leaves	3.5%	17.5%	46.0%	[64]
***Linum usitatissimum***	Seeds	43.4%	22.2%	3.3%	[65]
***Brassica napus***	Seeds	64.4%	19.7%	6.6%	[58]
***Arachis hypogaea***	Seeds	64.0%	18.0%	N/A	[51]
	Leaves	55.0%	17.0%	N/A	[51]

N/A: not available

**Table 6:** Numbers of *fad* genes in different species.

Species	*fad*2	References	*fad*3	References	*fad*6	References	*fad*7/8	References
***Artemisia sphaerocephala***	26	This work	3	This work	1	This work	9	This work
***Linum usitatissimum***	15	[53–55]	6	[55, 66, 67]	N/R	N/R	N/R	N/R
***Carthamus tinctorius***	11	[41]	1	[68]	1	[68]	2	[68]
***Glycine max***	5	[42–43]	4	[69–71]	2	[71]	4	[71]
***Brassica napus***	4	[48]	6	[48]	1	[72]	2	[72]
***Gossypium hirsutum***	4	[44–47]	3	[19]	N/R	N/R	3	[19]
***Helianthus annuus***	3	[49]	1	[73]	1	[73]	2	[73, 74]
***Arachis hypogaea***	3	[50–51]	1	[51]	1	[51]	1	[51]
***Arabidopsis thaliana***	1	[52]	1	[75]	1	[76]	2	[77, 78]

N/R: no report

**Fig 7:**
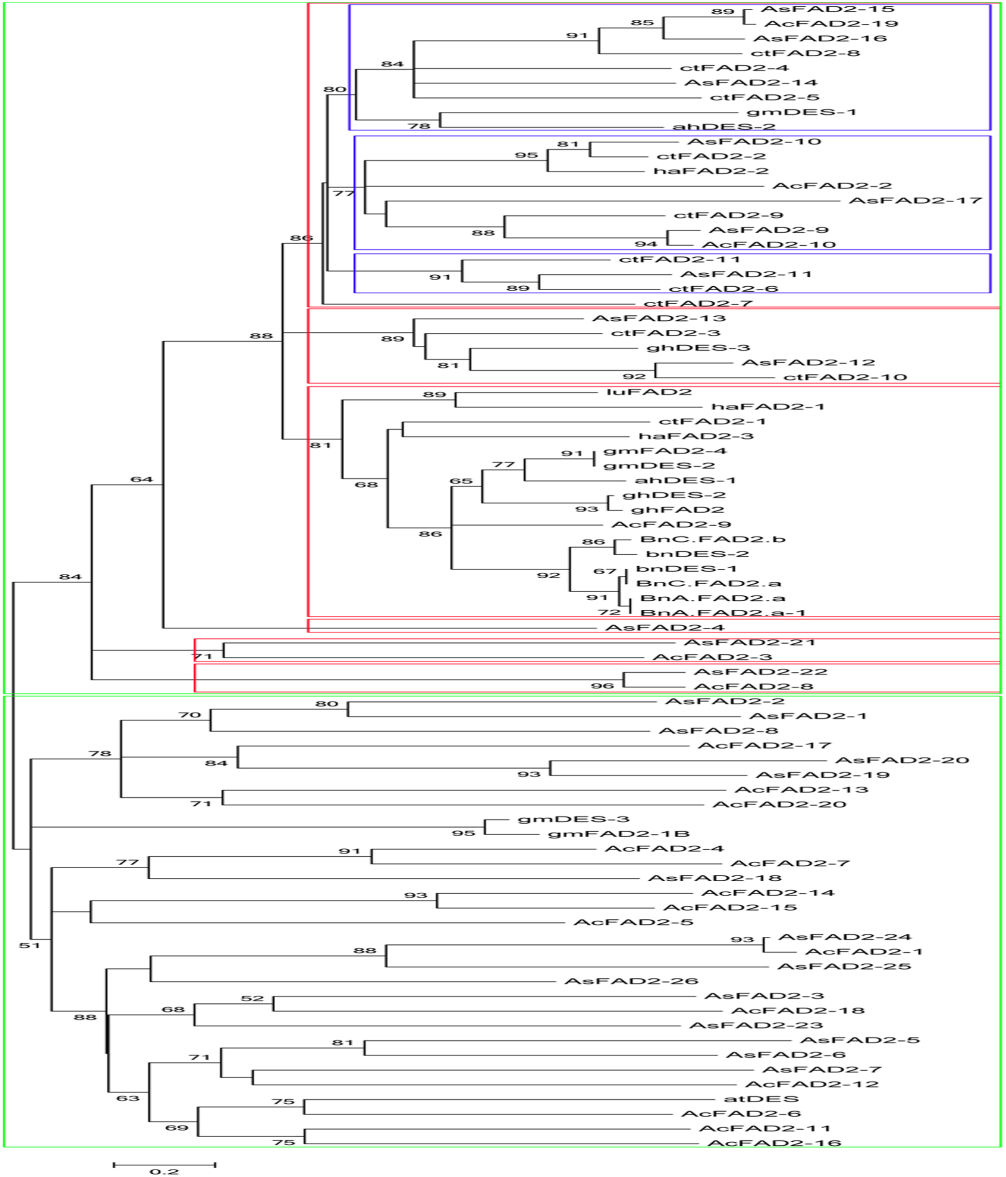
Phylogenetic comparison of *Artemisia sphaerocephala* AsFAD2s with FAD2s in other plants. Note: The phylogenetic tree was generated using Mega 5.0. The GenBank accession numbers of the amino acid sequences represented in the phylogenetic tree are as follows: CtFAD2-1, AGC65498.1; CtFAD2-2, AGC65499.1; CtFAD2-3, AGC65500.1; CtFAD2-4, AGC65501.1; CtFAD2-5, AGC65502.1; CtFAD2-6, AGC65503.1; CtFAD2-7, AGC65504.1; CtFAD2-8, AGC65505.1; CtFAD2-9; AGC65506.1; CtFAD2-10, AGC65507.1; CtFAD2-11, AGC65508.1; ghFAD2, AAQ16654.1; ghDES-2, AAL37484.1; ghDES-3, ADP02395.1; ahDES-1, ACZ06072.1; ahDES-2, AHN60569.1; gmFAD2-1B, ABF84062.1; gmFAD2-4, NP_001237865.1; gmDES-1, AAX29989.1; gmDES-2, AAB00860.1; gmDES-3, NP_001238342.1; BnA.FAD2.a-1, AFJ19029.1; BnA.FAD2.a, AFJ19030.1; BnC.FAD2.a, AFJ19031.1; BnC.FAD2.b, AFJ19032.1; bnDES-1, AGV77099.1; bnDES-2, AAT02411.1; haFAD2-1, AAL68981.1; haFAD2-2, AAL68982.1; haFAD2-3, AAL68983.1; luFAD2, AFJ53087.1; and atDES, AEE85834.1 (ct, *Carthamus tinctorius*; gh, *Gossypium hirsutum*; ah, *Arachis hypogaea*; gm, *Glycine max*; bn, *Brassica napus*; ha, *Helianthus annuus*; lu, *Linum usitatissimum*; at, *Arabidopsis thaliana*; As, *Artemisia sphaerocephala*; and Ac, *Artemisia annua*).

We employed ***fad* genes** from the transcriptomic data of *A*. *sphaerocephala* and those on oil crops, *A*. *thaliana* and two *Compositae* plants (*Artemisia annua* and *Carthamus tinctorius*) to further analyze the phylogenetic position of the *fad2* family, *Artemisia sphaerocephala* and *A*. *annua* protein sequences are shown in S5 Table. An evolutionary tree with 2 main categories (shown in green) was constructed (Fig 7), and the first category was divided into 6 parts (shown in red). Then, the first part was divided into 3 branches (shown in blue). AsFAD2-15, 16 and 14 are located on the first branch, and AsFAD2-15 and AcFAD2-19 are located next to each other and adjacent to AsFAD2-16 and ctFAD2-8. AsFAD2-14, ctFAD2-4 and ctFAD2-5 are also located on the first branch. The above results indicate that these genes might have high structural homology and similar functions. The first histidine motifs are HDCGHH in ctFAD2-5 and HECGHH in ctFAD2-4 and ctFAD2-8. ctFAD2-5 and ctFAD2-8 appear to be root specific, and ctFAD2-4 is highly expressed in young seedling tissues, including cotyledons and hypocotyls [68]. The second branch includes AsFAD2-10, 17 and 9. AsFAD2-10 and ctFAD2-2 are located next to each other; thus, we deduced that they have high structural homology and similar functions. ctFAD2-2 exhibited significantly increased expression in the cotyledonary tissues of young seedlings. The second histidine motif, HRRHH, is highly conserved in ctFAD2-2, which is a Δ12-oleate desaturase that converts oleic acid to linoleic acid and presumably plays only a secondary role in linoleic acid production in seed oil [68]. AsFAD2-9 was also situated next to AcFAD2-10 on the second branch. AsFAD2-11 and ctFAD2-6 are located next to each other on the third branch, and ctFAD2-6 has been found to be highly expressed in cotyledons and hypocotyls [68]. In addition, AsFAD2-12 and ctFAD2-10 are located next to each other in the second part. In ctFAD2-10, the amino acid immediately preceding the first histidine box is Ala (A); this gene is primarily expressed in flower tissues, with relatively low expression in other tissues, including the cotyledon, hypocotyls, root, and leaf [68]. AsFAD2-13 is also located in the second part next to ctFAD2-3. In CtFAD2-3, the serine at the +3 position is substituted by proline, which is also exclusively present in other FAD2 fatty acid conjugases [68]. The *fad2* genes of oil crops constitute the majority of the third part, and one sequence each from *A*. *annua* and *Carthamus tinctorius* are present in this part. However, no *A*. *sphaerocephala* sequence was observed in this part. AsFAD2-4 is located in the fourth part. AsFAD2-21 and AcFAD2-3 are located next to each other and form the fifth part. AsFAD2-22 and AcFAD2-8 are situated next to each other and form the sixth part. The second category contains AsFAD2-2, 1, 8, 20, 19, 18, 24, 25, 26, 3, 23, 5, 6, and 7. Genes in this category show high homology with genes in *A*. *annua*.

The *fad2* genes are members of a large and complex gene family, and they have base substitutions at multiple loci that result in amino acid differences [41]. Analysis of the evolutionary tree revealed that 26 putative FAD2s of *A*. *sphaerocephala* shared the highest homology with *A*. *annua*, and the second highest homology with sequences from safflower. There are 3 possible explanations for these results. First, *A*. *sphaerocephala* contains an abundance of linoleic acid; second, these three species belong to the *Compositae* family and have a close relationship; and third, the *fad2* gene family of safflower is the largest gene family discovered to date and serves as a significant reference. Interestingly, we found that FAD2s of traditional oil crops were located in the first category of the phylogenetic tree, particularly in the third group. We speculated that the conventional breeding process might have led to the loss of several genes from the wild species. In the second category of the tree, the *fad2* gene family of *A*. *sphaerocephala* showed obvious expansion and high homology with *A*. *annua*; FAD2s of these two species were predominant in this category and formed a very large *fad2* gene family.

Fatty acid desaturases are not only the key enzymes of unsaturated fatty acid synthesis but also have vital significance in terms of resistance to adverse environmental conditions. In a previous study, plant cold resistance was demonstrated to be reduced in an *A*. *thaliana fad2* mutant with a decrease in the unsaturated fatty acid concentration in extrachloroplast membrane lipids [79]. This *A*. *thaliana fad2* mutant has also been shown to accumulate a large amount of Na^+^ in the root cell cytoplasm and to be sensitive to salt stress during seed germination and early seedling growth [17]. Exogenous expression of sunflower *fad2* has been reported to increase the yeast unsaturated lipid index and membrane lipid fluidity, and resistance to salt and cold stresses was also enhanced [80]. The *fad3* gene is regulated through the synergistic and antagonistic interactions of plant hormones such as auxin, cytokinin, and abscisic acid, and the tissue specificity of the expression of this gene is further modified in accordance with the growth phase during plant development [81]. The *Arabidopsis* fad6 mutant exhibits obvious chlorosis at low temperatures [82]. In addition, FAD6 is required for salt resistance in *Arabidopsis* [83]. Further, overexpression of the *A*. *thaliana fad7* gene in tobacco seedlings during cryogenic treatment has been shown to result in etiolation [76, 84]. Studies have shown that the *fad7* gene is induced by light [85]. In *Arabidopsis*, fad8 expression is strongly promoted by low temperatures [77]. In addition, eleven *fad2* genes have been identified in a cDNA library constructed from 8 safflower tissues, including leaves, roots, cotyledons, hypocotyls, flowering heads, and developing embryos at 3 stages. The expression of *fad* genes is tissue specific [41, 42], and some of these genes display inducible expression [77, 85]. We constructed the *A*. *sphaerocephala* transcriptome by collecting 17 samples from plants grown under different conditions, including 4 stress-exposed samples, which could explain our finding of the largest *fad2* gene family to date in this organism.

### Candidate genes involved in stress responses in *Artemisia sphaerocephala*

**Fig 8:**
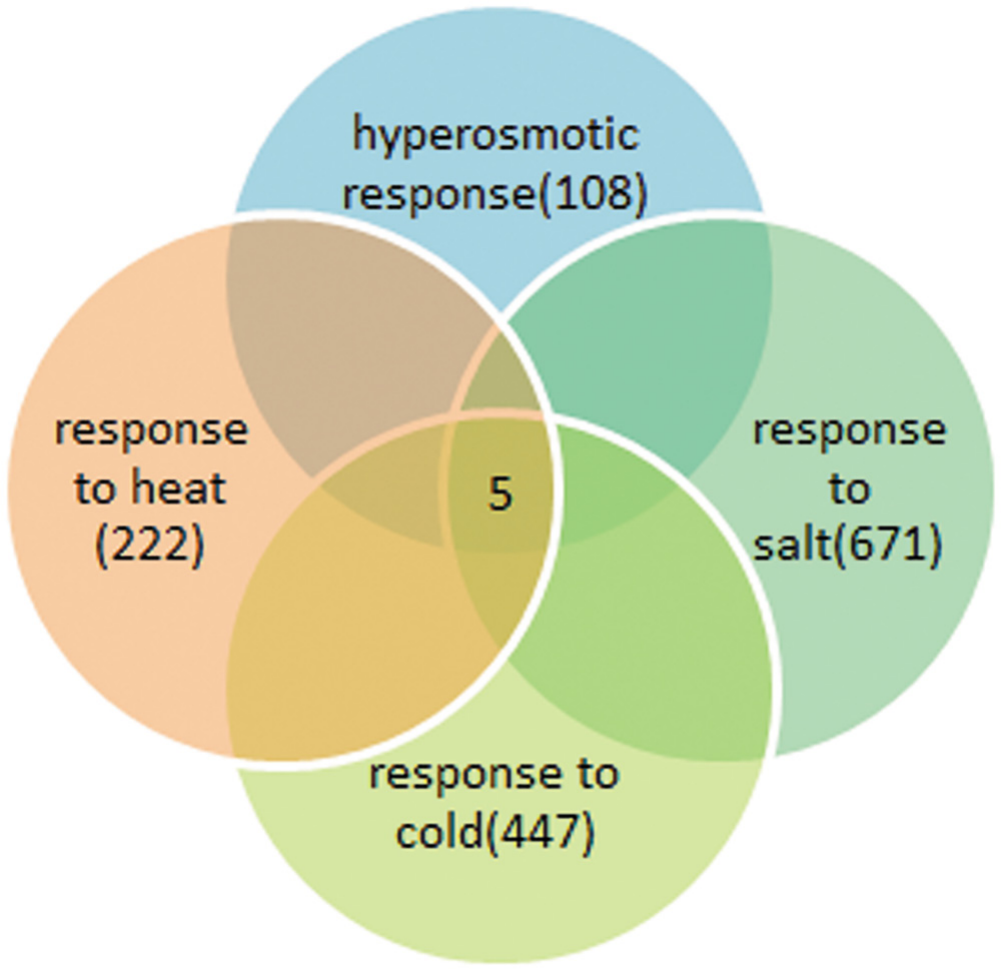
Venn diagram of stress-response unigenes.

**Fig 9:**
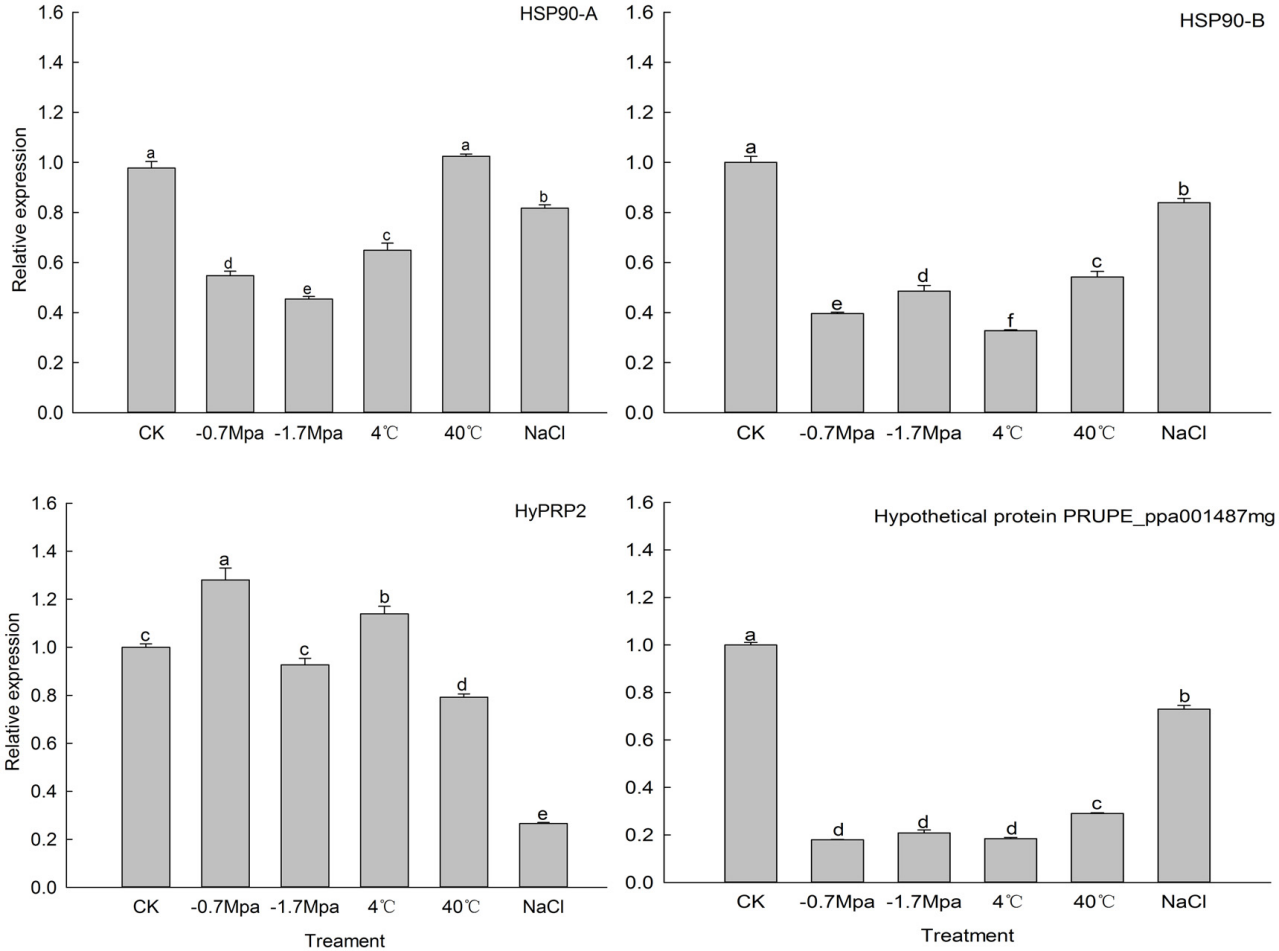
qRT-PCR analysis of stress-response genes.

Transcriptome analysis resulted in the identification of 1,212 unigenes involved in heat, cold, salt and drought responses. These 1,212 unigenes were annotated using GO terms, and 222, 447, 671 and 108 unigenes were found to have functions in heat, cold, salt and drought responses, respectively. Interestingly, 5 out of the 1,212 stress response unigenes, including 2 heat shock protein 90s (HSP90s), HyPRP2, the hypothetical protein PRUPE_ppa001487mg and an uncharacterized protein, play roles in all 4 biological response processes (Fig 8). Next, qRT-PCR was performed to verify the expression of these 5 genes in response to all four stress conditions. Four of these genes, excluding the uncharacterized protein, showed large expression differences compared with the control (Fig 9); no suitable primers could be designed for the uncharacterized protein because of its short sequence. Many studies have shown that HSPs accumulate under abiotic stresses, such as heat, cold, osmotic and salt stresses [86–90]. However, the expression levels of 2 HSPs in *A*. *sphaerocephala* were lower than those in the control under all treatments, with the exception of the expression of HSP90-A, which did not significantly differ between the 40°C treatment and the control. Recent studies have demonstrated that under normal conditions, HSP90 suppresses the functioning of heat shock factor (HSF); however, under heat stress, HSP90 is inactivated, and HSF is activated and regulates the expression of downstream genes to enable adaptation to oxidative stress [91–93]. Thus, low HSP90 expression may contribute to HSF activation in *A*. *sphaerocephala*. The expression levels of plant HyPRP genes vary in different species, in different organs within the same species, and in diverse environments [94–96]. Under cold, salt and drought stresses, cotton HyPRP expression is up-regulated [95]. Further, HyPRP overexpression decreases the tolerance of tobacco to cold and drought [96]. In this study, HyPRP2 expression was up-regulated under osmotic stress (-0.7 Mpa) and at 4°C, and it was down-regulated under salt stress and at 40°C. Expression of the hypothetical protein PRUPE_ppa001487mg was down-regulated under all treatments. The above results confirmed the expression changes of the stress response genes detected in transcriptome analysis. Expression profiling of the stress response genes must be carried out in a future study to elucidate the adaptive mechanisms of *A*. *sphaerocephala* to changing environmental conditions.

#### Heat tolerance

Daily and seasonal temperatures fluctuate greatly in the desert. A total of 222 unigenes classified as heat responsive were annotated using the Nr and Swiss-Prot databases (S6 Table). Of these unigenes, 64 potential chaperones, 55 putative enzymes, 15 transcription factors and 8 kinases were identified. Under the high temperature treatment, the plants exhibited a heat shock response (HSR), thereby triggering production of HSPs, which can act as molecular chaperones to enable the normal functioning of other proteins in high-temperature environments [97]. Therefore, the HSP family could improve the heat resistance of plants [98]. Four HSP110s, 7 HSP100s, 7 HSP90s, 14 HSP70s and 32 small HSPs have been discovered in *A*. *thaliana*, most of which are localized to the cytosol [99]. In the *A*. *sphaerocephala* transcriptome, 3 HSP110s, 5 HSP100s, 4 HSP90s, 4 HSP70s and 3 small HSPs were identified (S7 Table). Interestingly, 3 unigenes encoding putative HSPs, in addition to HSP90s, HSP70s and small HSPs were among the 1,000 most highly expressed unigenes (S8 Table), indicating that HSPs might contribute substantially to the high temperature resistance of *A*. *sphaerocephala*. One previous study has shown that a complex cascade mediates the up-regulation of HSPs and activation of heat shock transcription factors (HSFs), which show extremely essential function to regulate HSP gene expression [100]. *Arabidopsis thaliana* possesses diverse HSF families that include 21 genes [101, 102]. Fifteen HSFs were found in *A*. *sphaerocephala*, 5 of which had orthologs in *A*. *thaliana* (S9 Table). Fifteen unigenes out of the 1,000 most highly expressed unigenes were classified as heat responsive (S8 Table), including HyPRP2, a 17.7-kD class I small heat shock protein, inositol-3-phosphate synthase and others. Previous reports have shown that these genes are involved in processes used by plants for adaptation to external environments, especially hot environments [103–105]. These genes were highly expressed in *A*. *sphaerocephala* and might greatly contribute to the survival of this species in the hot desert environment.

#### Cold tolerance

Coldness has been proven to be one of the most significant factors limiting plant development. Temperatures of below zero during the winter can injure plant cells and tissues. Many plants are able to survive cold temperatures [106]. A total of 447 potentially cold-responsive unigenes were annotated using the Nr and Swiss-Prot databases (S6 Table). Among these unigenes, 13 potential chaperones, 6 late embryogenesis-abundant proteins (LEAs), 2 aquaporins, 222 enzymes, 8 transcription factors and 41 kinases were identified. The eight transcription factors, including the homeobox-leucine zipper protein (HD-zip), which is unique to the plant kingdom, are mainly involved in responses to abiotic stress and auxin, de-etiolation, and blue light signaling, as well as the regulation of organ growth and developmental processes [107–109]. Zinc finger proteins have been found to participate in extraordinarily diverse signal transduction pathways and developmental processes, including flower, seed and seedling development; trichome and root hair formation [110–113]; pathogen defenses [114]; and stress responses [115]. Zinc finger protein expression is increased in plants under both salt and cold stresses [116]. Excessive expression of zinc finger protein, which is induced by cold, enhances cold resistance in plants [117]. Dehydration-responsive element-binding protein/C-repeat binding factor (DREB/CBF) and a *cis*-acting element specifically bind to the dehydration-responsive element/C-repeat (DRE/CRT). The inducible production of DREB/CBF activates stress-resistant functions depending on the presence of DRE/CRT [106]. Two putative CBF/DEREs were discovered in *A*. *sphaerocephala*. WRKY transcription factors make up one of the largest families of transcriptional regulators in plants, and they form integral parts of signaling networks that mediate many plant processes [118]; some of these transcription factors were also classified as cold responsive in *A*. *sphaerocephala*. Among 447 cold-responsive unigenes, 52 were among the 1,000 most highly expressed unigenes, including HyPRP2, glycine-rich RNA-binding protein 7, 29-kD ribonucleoprotein and other highly expressed unigenes (S10 Table). These genes play important roles in resistance to cold stress [104, 117, 119].

#### Salt tolerance

High salt concentrations induce both osmotic and metal ion stresses. Plants have developed physiological and morphological strategies to adapt to these stresses [120]. To analyze the salt stress response and to screen for possible functional genes in *A*. *sphaerocephala*, 671 unigenes potentially responsive to salt stress were annotated using the Nr and Swiss-Prot databases (S6 Table). Of the 671 unigenes, 36 potential chaperones, 4 LEAs, 4 aquaporins and 286 enzymes, including reactive oxygen species (ROS) scavenging enzymes, MDAR, GST, superoxide dismutase (SOD) and catalase (CAT), were identified. In addition, 25 transcription factors and 58 kinases were found in the *A*. *sphaerocephala* transcriptome. The 25 transcription factors included bZIP (basic domain/leucine zipper), AP2/EREBP, HD ZIP, WRKY and MYB. Many studies have shown that these transcription factors play essential roles in the regulation of gene expression to cope with salt stress [121–125]. These transcription factors regulate and control the expression of downstream genes related to salt resistance by interacting with *cis*-acting elements. Extensive studies have shown that under high-salinity conditions, with the aid of Na^+^/H^+^ reverse transporters in the tonoplast, the expression of genes or the activities of H^+^-PPase and the vacuole-type H^+^-ATPase are increased, and Na^+^ enters into vacuoles [126–128]. Genes encoding Na^+^/H^+^ reverse transporters and vacuole-type H^+^-ATPase and H^+^-PPase were found in *A*. *sphaerocephala*. Salt overly sensitive (SOS) signal transduction pathwayis closely related to plant salt tolerance and has the most important role in ion homeostasis [129], A gene encoding ptotein involved in SOS pathway was also detected in *A*. *sphaerocephala*. Transcriptome analysis revealed that 103 out of 671 unigenes were among the top 1,000 highly expressed unigenes (S11 Table). Recent research has shown that HyPRP2, glycine-rich RNA-binding protein 7, glyceraldehyde-3-phosphate dehydrogenase, cysteine protease, HD-zip, isocitrate dehydrogenase, alcohol dehydrogenase 1 and other highly expressed unigenes participate in the process of salt tolerance and that expression of these genes improves the salt tolerance of plants [104, 130–133].

#### Drought tolerance

In desert regions, water deficit is an important factor affecting plant growth. To identify hyperosmotic response mechanisms and to screen for possible functional genes, 108 unigenes related to the hyperosmotic response were annotated using the Nr and Swiss-Prot databases (S6 Table). Among these unigenes, 17 kinases, 2 potential chaperones, 52 enzymes (including SOD), 6 transporters and channels and 3 aquaporins were identified. Although we did not identify transcription factors that were involved in the drought response, 25 out of the 108 unigenes were among the 1,000 most highly expressed genes (S12 Table), including HyPRP2, glycine-rich RNA-binding protein 7, glyceraldehyde-3-phosphate dehydrogenase, cysteine protease and others; thus, they might participate in the process of drought resistance and ensure normal plant growth in arid environments [104, 130–132].

#### Multiple stresses

Recurrent or multiple environmental stresses, such as high solar radiation and extreme temperatures, occur in the desert, and high evaporation rates lead to vapor pressure deficits and thus water stress. Salt also accumulates in surface soil. Combinations of various abiotic stresses affect plants simultaneously. Recent plant transcriptome studies have shown that the molecular mechanisms related to multiple stresses might differ from those associated with a single stress [134–137]. A total of 190 specific candidate genes are necessary for plant responses to various combinations of stresses, including salinity, osmotic stress and high temperature [136]. We found that 58 out of these 190 genes had orthologs in *A*. *sphaerocephala* (S13 Table) and that 8 of these unigenes were included among the 1,000 most highly expressed unigenes. These genes help *A*. *sphaerocephala* to minimize stress-related damage and to protect itself in harsh environments.

To reduce the chance of mistaking a paralog for an ortholog, we identified pairs of putative orthologs consisting of reciprocal best hits (RBHs) [138]. This approach resulted in detection of 17,904 pairs of putative orthologs that each corresponded to a single *A*. *sphaerocephala* unigene and *A*. *thaliana* peptide sequence (S14 Table). We compared 1,212 unigenes with 17,904 pairs of putative orthologs and identified 548 unigenes in *A*. *thaliana*, including 23 transcription factors, 6 LEAs, 2 aquaporins, 29 chaperones, 229 enzymes and 35 kinases. Among the 23 transcription factors, HD-zips and zinc finger proteins were classified as cold responsive, and 16 transcription factors, including MYB, WRKY, and bZIP transcription factors, were classified as salt responsive. Further, 5 HSFs were found to function in response to heat stress, and 2 aquaporins were determined to function in response to cold and salt stresses. Among the 6 LEAs, 4 were classified as cold responsive, and 4 were classified as salt responsive. The other 664 unigenes were also found to play important roles in stress responses in *A*. *sphaerocephala*, and they included 25 transcription factors, 3 LEAs, 2 aquaporins, 62 chaperones, 359 enzymes and 60 kinases. Among the 25 transcription factors, 9 HSFs were found to function in response to heat, and 4 transcription factors were determined to function in response to cold, including HD-zips and WRKY transcription factors. Further, 12 transcription factors were classified as salt responsive, including bZIP and MYB transcription factors, 3 LEAs were classified as cold and salt responsive, and 2 aquaporins were classified as cold, drought and salt responsive.

## Conclusions and Perspectives

*Artemisia sphaerocephala* is a desert plant species with high ecological and economic value. In this study, 68,373 *A*. *sphaerocephala* unigenes were identified by high-throughput sequencing using an Illumina HiSeq 2500 platform, 58.7% of which were annotated. A set of heat, cold, salt and drought stress-responsive genes were identified, as well as 26 *fad2*, 3 *fad3*, 1 *fad6*, and 9 *fad7/8* genes. To our knowledge, this study is the first to use high-throughput sequencing to investigate the global transcriptome of *A*. *sphaerocephala*. This study has provided valuable genetic resources that may be useful for modification of plant unsaturated fatty acids. These genetic findings are also potentially very valuable for increasing our understanding of the unsaturated fatty acid synthesis pathway, the biological effects of *fad* genes on stress and the molecular adaptive mechanisms of desert plants.

## Supporting Information

S1 Fig Histogram of the length distribution of transcripts. (DOCX)

S2 Fig Histogram of the length distribution of ORFs. (DOCX)

S3 Fig Nr annotation results distributed by E-values. (DOCX)

S4 Fig Length distributions of annotated and un-annotated unigenes according to log(length). (DOCX)

S5 Fig Expression levels of annotated and un-annotated unigenes according to log(FPKM). (DOCX)

S6 Fig The top 50 represented GO terms. (DOCX)

S7 Fig Nr annotation results based on sequence identities. (DOCX)

S1 Table Information for 5 stress-response genes. (XLSX)

S2 Table Primer information for 5 stress-response genes. (DOCX)

S3 Table Length distribution of open reading frames (ORFs). (DOCX)

S4 Table Summary of the final *Artemisia sphaerocephala* transcriptome annotation. (XLSX)

S5 Table *Artemisia sphaerocephala* and *Artemisia annua* FAD2 protein sequences. (XLSX)

S6 Table Annotation of stress response unigenes using the Nr and Swiss-Prot databases. (XLSX)

S7 Table HSPs in the *Artemisia sphaerocephala* transcriptome. (XLSX)

S8 Table Highly expressed heat-responsive unigenes. (XLSX)

S9 Table Fifteen HSFs in *Artemisia sphaerocephala*. (XLSX)

S10 Table Highly expressed cold-responsive unigenes. (XLSX)

S11 Table Highly expressed salt-responsive unigenes. (XLSX)

S12 Table Highly expressed hyperosmotic—responsive unigenes. (XLSX)

S13 Table Possible genes involved in multiple stress responses. (XLSX)

S14 Table Pairs of putative orthologs between *Artemisia sphaerocephala* and *Arabidopsis thaliana*. (XLSX)
