# Supplementary figures and images for: Genome-Scale Transcriptome Analysis of the Desert Shrub Artemisia sphaerocephala
Source: PLoS One. 2016 Apr 26;11(4):e0154300. doi: 10.1371/journal.pone.0154300 (PMC4846011; doi:10.1371/journal.pone.0154300)

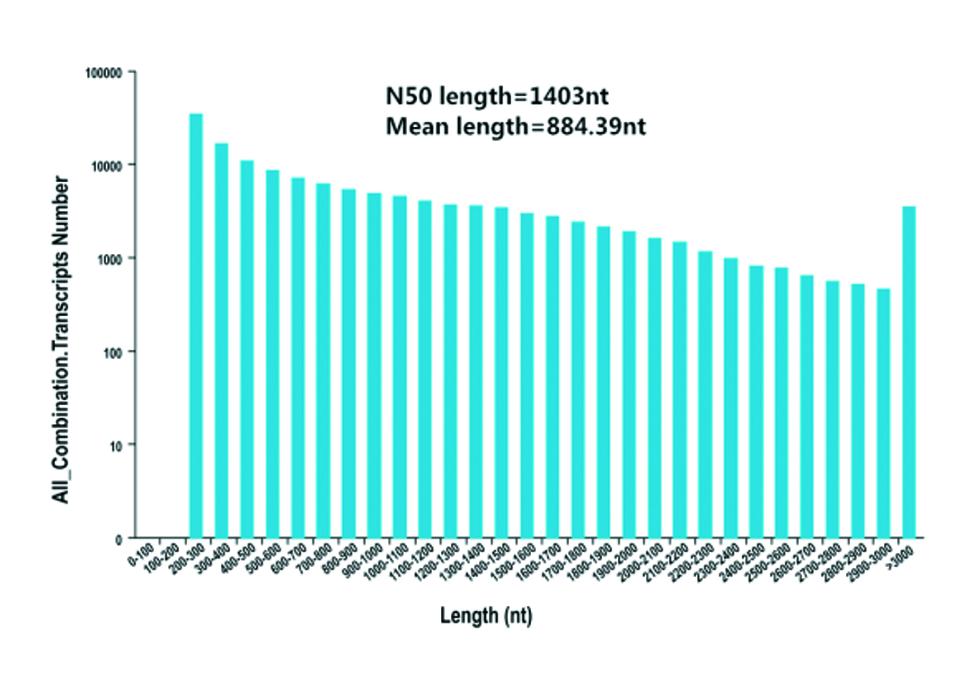


S1 Fig. Histogram of the length distribute of transcripts.

Supplement: S1 Fig — (DOCX) [file pone.0154300.s001.docx]

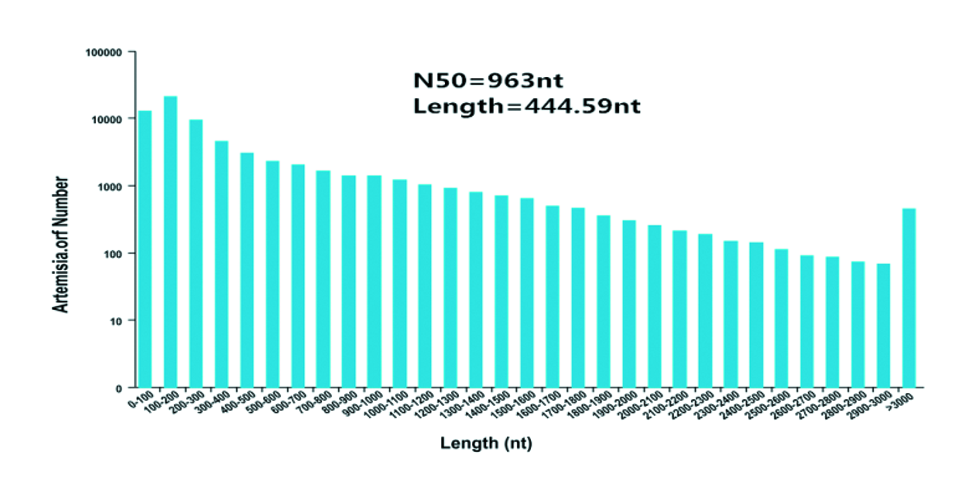


S2 Fig. Histogram of the length distribute of ORFs.

Supplement: S2 Fig — (DOCX) [file pone.0154300.s002.docx]

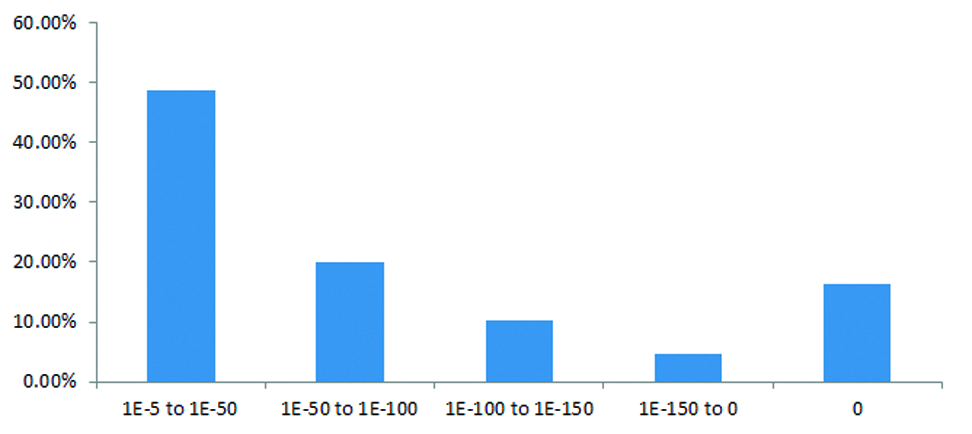


S3 Fig. Nr annotation results distributed by the E-value.

Supplement: S3 Fig — (DOCX) [file pone.0154300.s003.docx]

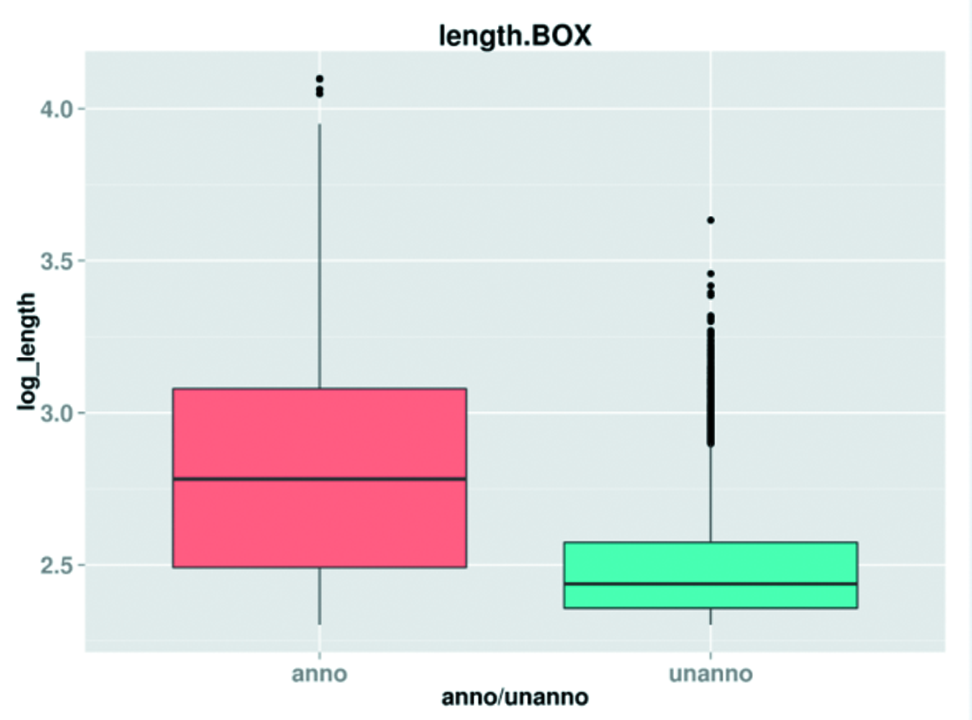


S4 Fig. Length distribution of annotated and un-annotated unigenes was inferred with log (Length).

Supplement: S4 Fig — (DOCX) [file pone.0154300.s004.docx]

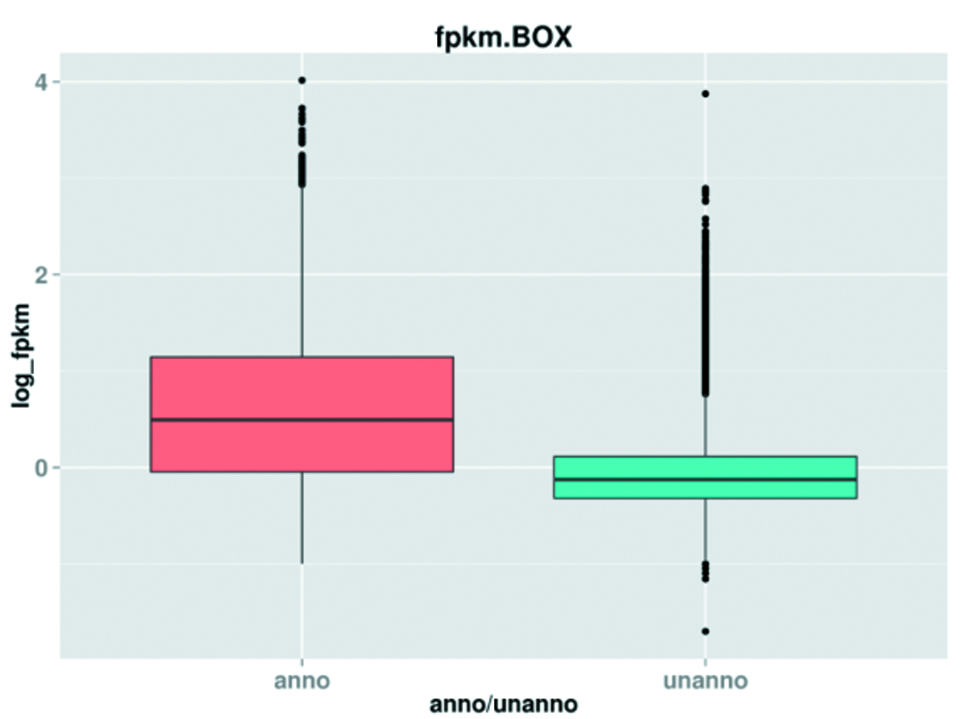


S5 Fig. Experssion level of annotated and un-annotated unigenes was inferred by log (FPKM).

Supplement: S5 Fig — (DOCX) [file pone.0154300.s005.docx]

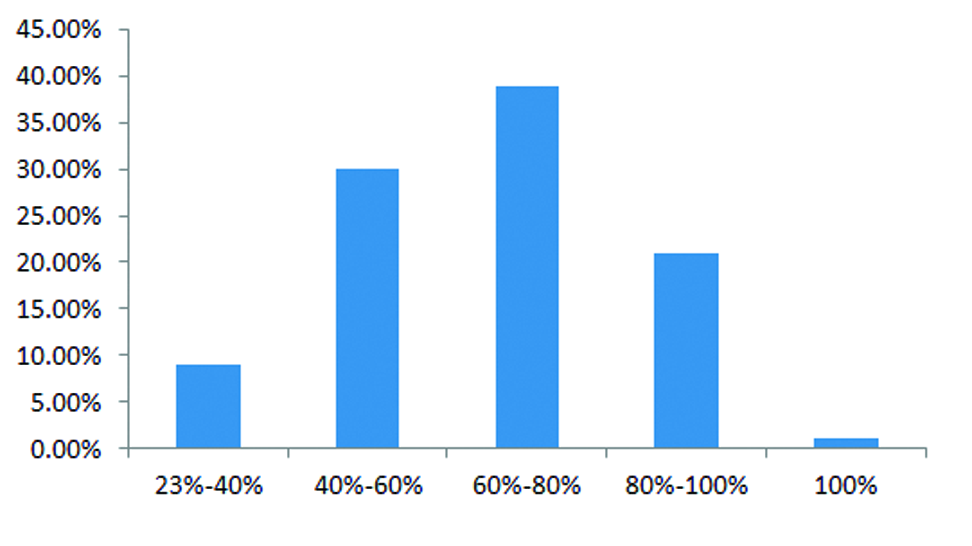


S7 Fig. Nr annotation results based on sequence identities.

Supplement: S7 Fig — (DOCX) [file pone.0154300.s007.docx]
