# Supplementary material for: Genome-Scale Transcriptome Analysis of the Desert Shrub Artemisia sphaerocephala
Source: PLoS One. 2016 Apr 26;11(4):e0154300. doi: 10.1371/journal.pone.0154300 (PMC4846011; doi:10.1371/journal.pone.0154300)
Supplement: S6 Fig — (DOCX) [file pone.0154300.s006.docx]

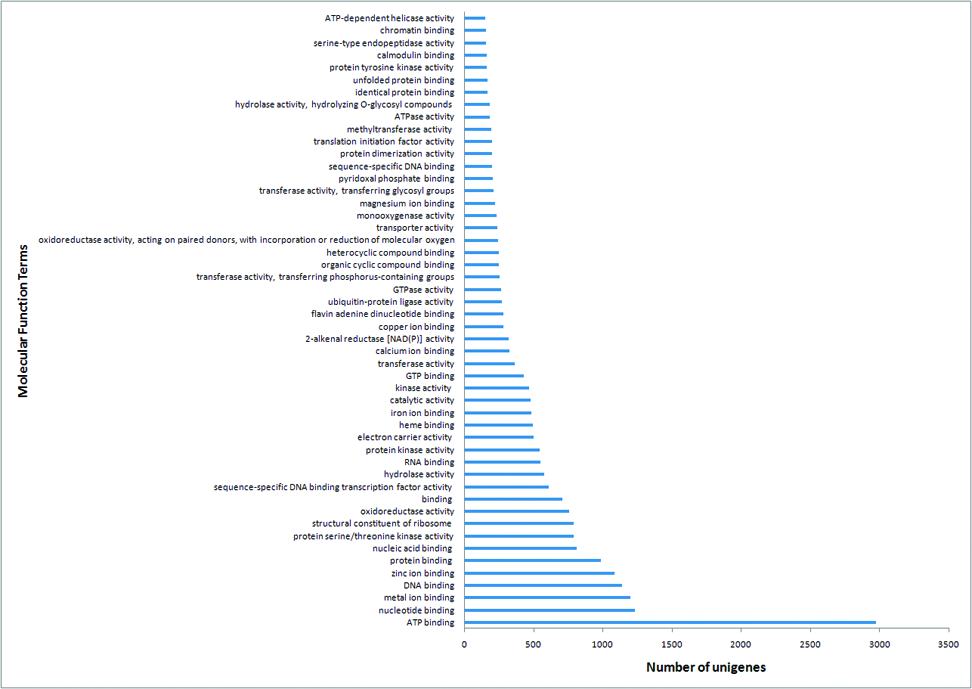


S6-1 Fig. The top 50 represented MF GO terms of unigenes.


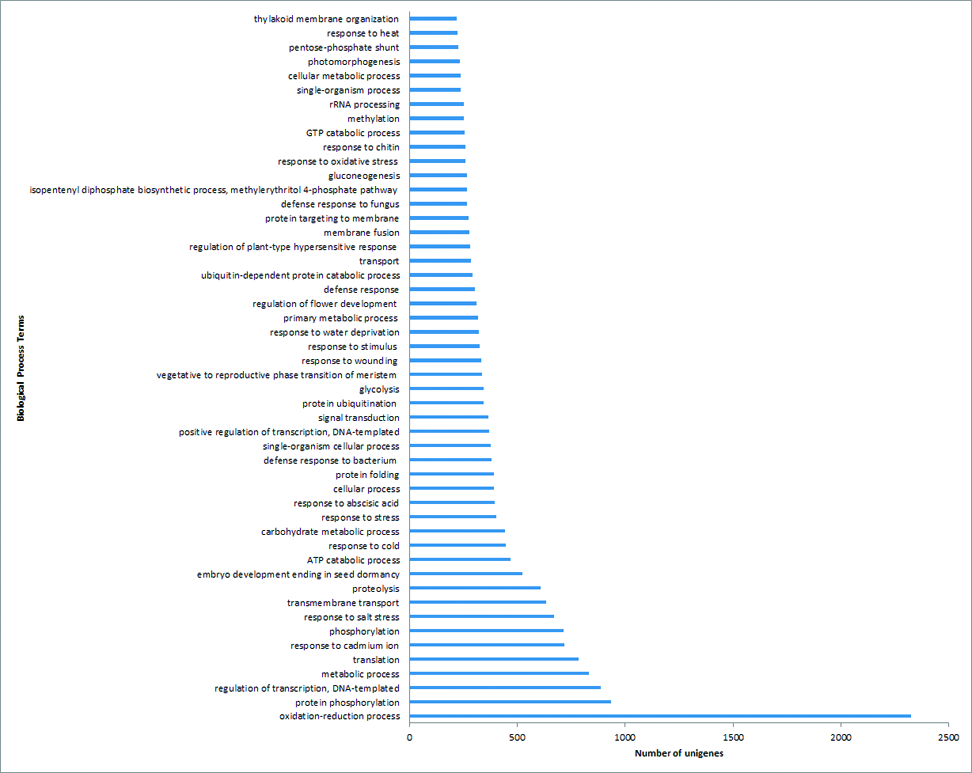


S6-2 Fig. The top 50 represented BP GO terms of unigenes.


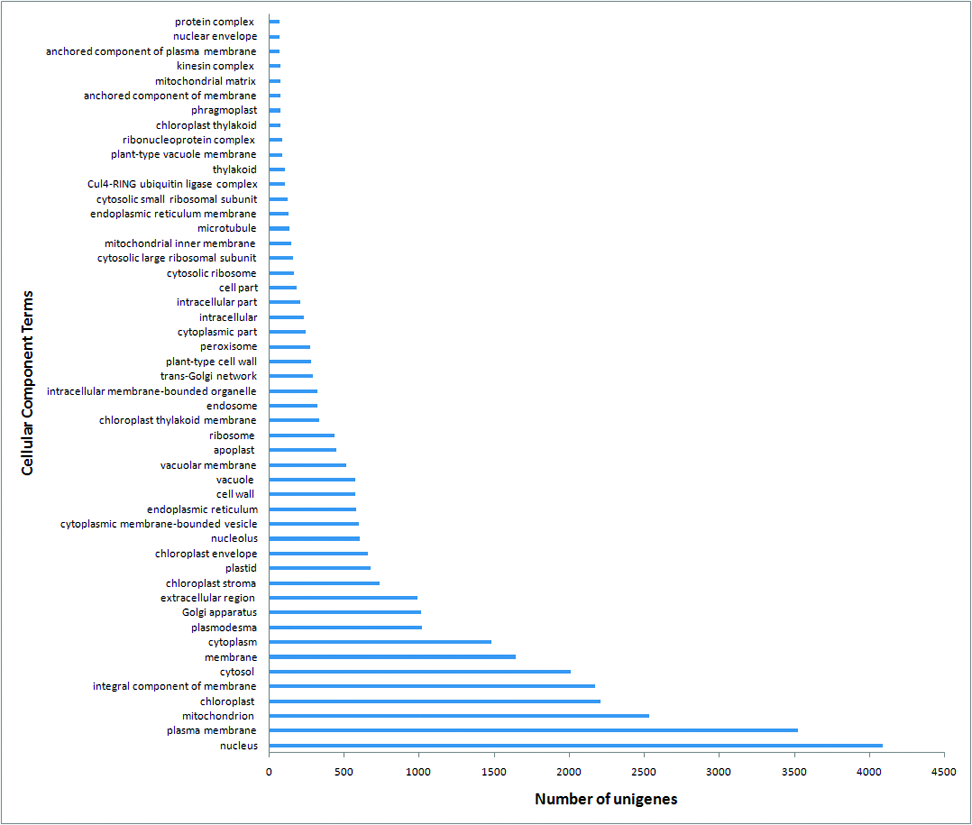


S6-3 Fig. The top 50 represented CC GO terms of unigenes.
