# Supplementary material for: Genome-Scale Transcriptome Analysis of the Desert Shrub Artemisia sphaerocephala
Source: PLoS One. 2016 Apr 26;11(4):e0154300. doi: 10.1371/journal.pone.0154300 (PMC4846011; doi:10.1371/journal.pone.0154300)
Supplement: S2 Table — (DOCX) [file pone.0154300.s009.docx]

| Gene name | Primer pair (forward/reverse) | Product size (bp) |
| --- | --- | --- |
| HSP90-A | GGTCCAAACCTAATGCGAGT | 122 |
|  | CAGCCCAAAGGAAGAAACTG |  |
| HSP90-B | CACAGAACGGCTTTGAACTC | 100 |
|  | TAGTGGGTGTTTTCGTATGTCAG |  |
| HyPRP2 | TGAAACTCCTACCCCCACAC | 130 |
|  | TGAAACCAATCCGCCAAG |  |
| Hypothetical protein | TGAGTTCAAGGCCGTTCTG | 136 |
|  | TGAAGCATGTCACGAGAAACA |  |

S2 Table. Primer information of 5 stress-response genes.
