# Supplementary material for: Genome-Scale Transcriptome Analysis of the Desert Shrub Artemisia sphaerocephala
Source: PLoS One. 2016 Apr 26;11(4):e0154300. doi: 10.1371/journal.pone.0154300 (PMC4846011; doi:10.1371/journal.pone.0154300)
Supplement: S3 Table — (DOCX) [file pone.0154300.s010.docx]

S3 Table. Length distribution of Open Reading Frames (ORFs).

| ORF Length | Total Number | Percentage |
| --- | --- | --- |
| 0-300 | 43018 | 63.34% |
| 300-500 | 7512 | 11.06% |
| 500-1000 | 8724 | 12.85% |
| 1000-2000 | 6851 | 10.09% |
| 2000+ | 1812 | 2.67% |
| Total Number | 67917 |  |
| Total Length | 29923746 |  |
| N50 Length | 963 |  |
| Mean Length | 440.5928707 |  |
